# Exploring Unprofessional Behaviors and Biased Perceptions in the Clinical Environment: Students’ Perspectives

**DOI:** 10.1007/s40670-024-02087-9

**Published:** 2024-06-08

**Authors:** Xiaomei Song, Mildred J. Willy

**Affiliations:** 1 School of Medicine, Case Western Reserve University, 9501 Euclid Ave., Cleveland, OH 44106 USA; 2 College of Medicine, Central Michigan University, Mount Pleasant, MI USA

**Keywords:** Professionalism, Bias, Medical students, Clinical setting, Mixed methods

## Abstract

Professionalism stands as a fundamental cornerstone within the realm of physician training, representing a core competency that holds significant importance. It entails creating workplaces that are physically and psychologically safe within the health care system. Positive role modeling from health professionals is important in creating a learning environment that fosters diversity, equity, and inclusion for all. Using the mixed-methods sequential design, this study investigated unprofessional behaviors and biased perceptions demonstrated by health professionals as perceived and experienced by medical students during their clinical rotations at one medical school. Seventy-three M3 students and 37 M4 students responded to the survey, followed by three focus groups (*n* = 11) to further examine unprofessionalism and biases as well as impacts on performance, learning opportunities, and well-being. The results from both the quantitative and qualitative data demonstrate the presence of unprofessionalism and biases within the current medical education environment. These issues include a lack of respect and compassion, a lack of commitment to professional duties, malfunctioning teamwork, and a lack of sensitivity towards individuals regardless of their group affiliations. The unprofessional behaviors and biased perceptions have detrimental impacts on students’ performance, learning, and well-being. The reasons behind unprofessionalism and bias are multifaceted, influenced by societal and local environmental factors that extend beyond individual beliefs and values. By collecting empirical data on students’ experiences and perceptions, the study sheds light on the areas that need improvement and offers insights into adopting strategies to decrease unprofessional conducts and foster a respectful and inclusive learning environment.

## Introduction

The medical school learning environment, which encompasses various physical, social, and psychological elements, collectively influences how students develop both academically and professionally [1, 2]. The establishment of psychological safety and role modeling by health professionals plays a pivotal role in cultivating a positive learning environment. Such an environment addresses unprofessional behaviors; recognizes and mitigates bias; prioritizes diversity, equity, and inclusiveness; and effectively nurtures a growth mindset and cultural competence [3]. Professionalism stands as one of the fundamental core competencies in the training of physicians. The importance of professionalism is widely recognized because it directly impacts patient care and safety, as well as the overall reputation and trustworthiness of the medical profession by society. Although professionalism has been extensively defined and examined, professionalism does not have a set, universal definition which contributes to inconsistency of behaviors. The ACGME included professionalism as a competency for residents in 1999 [4]. The Physician Competency Reference Set (PCRS) defines professionalism as a set of attributes and behaviors. [5] The AAMC graduate questionnaire specifically assesses negative behaviors and professionalism in the learning environment for each medical school with annual comparative data using Likert-scale questions. As such, it is important to investigate whether healthcare workers demonstrate and role model professional attributes and behaviors when interacting with patients, working with colleagues, and teaching and mentoring students. Included in this is the need to investigate how unprofessional behaviors and bias experienced by learners may influence students’ learning, performance, and well-being. The purpose of this study is to gain a deeper insight into negative behaviors and bias exhibited by healthcare workers as perceived and encountered by medical students during M3 and M4 clinical rotations at one medical school.

## Professionalism in the Context

The literature discusses various unprofessionalism and bias faced when experiencing disparities in the quality of healthcare. The behaviors and attitudes of healthcare providers were identified as one of many factors [6]. In medical education, the research found that gender bias impacted faculty assessment of medical student learners [7]. Brown et al. [8] used a qualitative, constructivist approach to investigate bias based on the experiences of medical students from multiple UK medical schools. A systematic review conducted by FitzGerald and Hurst concluded that healthcare professionals exhibited the same levels of implicit bias as the wider population and highlighted the need for the healthcare profession to address the role of implicit biases in disparities in healthcare [9]. Healthcare disparities can arise due to various factors such as race, gender, ethnicity, socioeconomic status, age, and other marginalized identities. Understanding and addressing these issues are crucial for achieving equitable healthcare outcomes for all individuals and improving the training of culturally competent physicians. Investigations of unprofessionalism and biased perceptions are crucial for addressing disparities and fostering an inclusive learning environment.

While there is general agreement within the medical education community about the significance of professionalism, it is acknowledged that a one-size-fits-all approach does not suit all learning contexts when determining the specific knowledge, skills, and attitudes essential for demonstrating professional conduct [10–12]. The definition of professionalism is inherently contextual, and its definition is significantly shaped and influenced by the various features of the local context [13]. The local context encompasses a range of factors, including contextualized cultural/clinical norms, societal expectations, institutional policies, and professional standards that influence the understanding and definition of professionalism. Likewise, medical schools adopt various curriculum and context-specific instructional methods to develop student competency in professionalism. These methods range from dedicated courses on professionalism, interactive series of workshops, faculty or peer feedback, and written reflection on professionalism challenges to personalized coaching and mentoring [14, 15]. Needless to say, the unspoken, hidden curriculum, often conveyed through implicit messages, norms, and cultural dynamics within the learning environment, influences students’ understanding of professionalism [16, 17]. Within the context, faculty and clinicians serve as influential role models, significantly shaping students’ professional identity and values. Through their everyday actions, interactions, and attitudes, healthcare professionals exemplify the standards of professional conduct expected in the field.

The Central Michigan University College of Medicine (CMED) is dedicated to educating a diverse student body and preparing culturally competent physicians to serve underserved populations in Michigan and beyond, as outlined in its mission statement. Guided and influenced by the PCRS, with some modifications tailored to the CMED context, the educational program’s objectives, including the domain of professionalism, have been in effect since 2013. This domain requires students to demonstrate “a commitment to carrying out professional responsibilities, adherence to ethical principles, and sensitivity to all individuals.” The specific competency encompasses a wide range of attributes, including (1) demonstrating respect and compassion; (2) demonstrating accountability, integrity, and a commitment to excellence; (3) respecting patient’s privacy and autonomy; (4) demonstrating sensitivity and responsiveness to all individuals; (5) enhancing team functioning; (6) demonstrating a commitment to ethical principles; and (7) giving and receiving candid and constructive feedback. While professionalism has been broadly used as an overarching concept at CMED, bias is specifically defined as the absence of sensitivity and responsiveness to all individuals, irrespective of gender, age, culture, race, religion, ability, sexual orientation, socio-economic status, or medically underserved status. Due to its significant impact on student subgroups, bias has received special attention at CMED.

CMED embraces a range of policies, with its professionalism and technical standards firmly grounded in the physician charter, which was developed and revised by professional organizations [18, 19]. Examples of expectations are included in the student handbook and provided to each medical student. Furthermore, the CMED curriculum related to professionalism includes various learning objectives, instructional activities, and assessment tools such as orientation sessions, peer assessments in years 1–2 for their small groups, ongoing training sessions, clerkship goals and objectives, and work-based professionalism clinical assessment. The CMED Curriculum Committee oversees the development and effectiveness of curriculum activities and outcomes related to professionalism. The CMED Learning Environment Committee plays an important role in performing periodic assessments and monitoring of the learning environment to address systematic or institutional cultural issues that serve as barriers to the optimal learning environment. The school and its clinical affiliates share the responsibility for periodic overall evaluation of the learning environment to promptly correct violations of professional standards, identify positive and negative influences on the maintenance of professional standards, and develop and conduct appropriate strategies to enhance positive and mitigate negative influences.

Using the mixed-methods explanatory sequential design, this study investigated unprofessional behaviors and biased perceptions demonstrated by health professionals as perceived and experienced by medical students during their clinical rotations at one medical school. The findings of the study can provide valuable rationales for implementing changes within the medical education system. By collecting empirical data on students’ experiences and perceptions, the study sheds light on the areas that need improvement and highlights specific actions or attitudes that may undermine the learning environment or impact students’ experiences. Specifically, the study aimed to understand and address the following research questions:

1. Did students experience unprofessionalism and biases in the clinical setting? If so, in what ways?
2. Do students perceive that unprofessional behavior and biases in the clinical setting make impacts on their learning, performance, and well-being? If so, in what ways?

## Methods

Using a mixed-methods sequential design, we first collected and analyzed quantitative data through the items in the learning environment survey followed by focus groups via WebEx. While the quantitative stage assesses descriptive information and statistical significance, the qualitative stage explains the phenomena with an in-depth scope and range of inquiries [20]. The quantitative data collection and analyses were completed before the qualitative stage. In this study, the results of the quantitative data were used to inform the design of the semi-structured interview protocol during the qualitative stage, which had a broader scope. Therefore, the qualitative stage was put more weight, providing depth to explain the initial quantitative results and expanding our understanding of the research questions asked. The results of the two stages were merged in the discussion.

In this study, the quantitative part primarily focused on the areas closely related to professionalism within the learning context. The CMED Learning Environment Committee designed the learning environment survey, informed and modified based on the AAMC Graduation Questionnaire, the Geisinger Commonwealth School of Medicine Learning Environment Survey (accessed through personal connections), and the literature [21–23]. In addition, the Student Diversity Committee and all student groups representing diversity at the school reviewed the survey and provided feedback. The survey items related to professionalism included two parts: (1) disconnection and unprofessional behaviors demonstrated by faculty, residents, and nursing and (2) perceived bias based on ethnicity, gender, etc. in clinical assessments and learning opportunities and the effect on learning and well-being. All the M3 and M4 students were invited to complete the survey, and responses were analyzed using SPSS.

After all the quantitative data in the learning environment survey were analyzed and interpreted, the findings of the survey led to the Learning Environment Committee’s decision to collect additional information related to professionalism from focus groups. We were requested by the learning environment committee to further examine and understand descriptions specific to unprofessional behavior and bias demonstrated by faculty. As such, the qualitative stage probed students’ interactions more broadly and explored a larger learning context. Based on the quantitative findings, a semi-structured interview protocol (see Appendix) was designed asking students to provide their interpretations of survey results and personal experiences by giving specific examples. Significant efforts were undertaken to ensure confidentiality and adequate representation. Eventually, only nine female and two male volunteers were recruited to explore students’ perceptions in depth. Three focus groups were conducted, and all focus groups were conducted by one research team member who did not directly interact with students in the educational program. The participants were provided with CMED’s professionalism competency prior to the interview. Focus group interviews were recorded and transcribed verbatim, and the transcriptions were analyzed using a thematic inductive coding method. Two researchers developed the initial draft coding scheme, followed by an iterative process of modifying and recoding before producing a finalized codebook.

## Results

### Quantitative Findings

Overall, among all the M3 (*n* = 101) and M4 (*n* = 98) students, 73 M3 students (72%) and 37 M4 (38%) students responded to the survey. Thirty-seven percent M3 and 30% M4 participants reported that they frequently or often observed disconnects between the level of professionalism demonstrated by faculty and what they were taught about professionalism. Approximately 3% of M3 and 6% of M4 medical students reported they frequently or often experienced unprofessional behaviors exhibited by their peers. Similarly, 1% of M3 students and 5% of residents were reported to demonstrate such behaviors, and only 3% of M3 students reported experiencing unprofessional behaviors from nursing staff. Furthermore, 20% of M3 students and 11% of M4 students disagreed or strongly disagreed that their clinical assessments were solely based on their knowledge, not influenced by other factors.

The participants shared numerous accounts of explicit and implicit bias related to race, gender, religion, ethnicity, and socioeconomic status. Among the 73 M3 students, 7 reported experiencing explicit bias based on race, followed by 3 who mentioned instances related to religion, 2 regarding ethnicity, and 2 related to gender. Ten M3 students reported encountering implicit bias based on race, followed by 7 who discussed bias related to gender, 4 regarding ethnicity, 2 related to religion, and 2 related to socioeconomic status.

In the 37 M4 group, one M4 student reported explicit bias based on gender, and another reported explicit bias based on race. Additionally, 2 M4 students mentioned experiencing implicit bias based on gender, 1 related to socioeconomic status, and 1 related to weight (Table 1).

**Table 1:** Descriptive statistics

	M3 (response rate 72%)					M4 (response rate 38%)				
	Frequently	Often	Sometimes	Rarely	Never	Frequently	Often	Sometimes	Rarely	Never
There are disconnects between what I am taught about professional behaviors and the level of professionalism I see being demonstrated by faculty	16% (12)	21% (15)	32% (23)	30% (22)	1% (1)	8% (3)	24% (9)	19% (7)	24% (9)	24% (9)
I have experienced unprofessional behavior demonstrated by my fellow students at CMU College of Medicine	3% (2)	0% (0)	41% (30)	29% (21)	27% (20)	3% (1)	3% (1)	27% (10)	41% (15)	27% (10)
I have experienced unprofessional behavior demonstrated by the residents at CMU College of Medicine	0% (0)	1% (1)	16% (12)	16% (12)	66% (48)	0% (0)	5% (2)	11% (4)	35% (13)	49% (18)
I’ve experienced unprofessional behavior demonstrated by my nursing at CMU College of Medicine	0% (0)	3% (2)	23% (17)	14% (10)	60% (44)	0% (0)	0% (0)	24% (9)	30% (11)	46% (17)
	Strongly Disagree	Disagree	Neutral	Agree	Strongly Agree	Strongly Disagree	Disagree	Neutral	Agree	Strongly Agree
I feel that clinical assessments are based solely on my knowledge and performance and are not influenced by other factors, such as gender, race, age, etc	8% (6)	12% (9)	23% (17)	39% (28)	18% (13)	0% (0)	11% (4)	16% (6)	32% (12)	41% (15)

	M3 (response rate 72%)						M4 (response rate 38%)				
	Ethnicity	Gender	Race	Religion	Socioeconomic Status	None	Gender	Race	Weight	Socioeconomic Status	None
I sense there is explicit bias at CMU College of Medicine based on one or more of the following	2	2	7	3	0	59	1	1	0	0	35
I sense there is implicit bias at CMU College of Medicine based on one or more of the following	4	7	10	2	2	48	2	0	1	1	33

### Qualitative Findings

During the focus group interview, the participants were given the chance to expand on their personal experiences, provide vivid examples, delve deeply into their reflections, and explore various aspects related to professionalism. Three themes emerged from focus groups: (1) key features of unprofessionalism and bias, (2) potential causes of unprofessionalism and bias, (3) impacts on learning and well-being.

#### Key Features of Unprofessionalism and Bias

Regarding the key features of unprofessionalism and bias, there were four major codes: a lack of respect and compassion, a lack of commitment to professional responsibilities, malfunctioning teams, and a lack of sensitivity to all individuals regardless of group status. First, during the interviews, the participants shared their experiences and perceptions of how faculty members, staff, and health professionals demonstrated a lack of respect and compassion. They described how faculty members were “sarcastic” and came off the wrong way, mispronounced students’ names repeatedly, were discouraging by telling students they were not going to match, and at times staff members were “condescending” and gave half information or buried information. The following student provided an elaboration of personal experience when the patients were placed under anesthesia:

Once they are put under anesthesia it is a little dehumanizing and often times kind of concerning coming from nursing staff and the attending…The comments that are made about the patients, just because they're asleep, are quite unprofessional and something that I wouldn't want to hear from an attending or someone who's in the healthcare field [taking care of me]. Um, So, coming from someone who's, I'm interested in surgery, it was quite sad to see the team... It's kind of eye-opening and very sad to watch. (P2)

At CMED, clinical faculty members hold faculty appointments and residents undergo training programs specifically designed to prepare them for their role as teachers. Although attendings and residents were required to fulfill their obligations in student teaching and assessment, not every attending physician and resident consistently demonstrated accountability and commitment to teaching medical students. The participants described how attendings did not respond to students’ texts repeatedly and “wanted nothing to do with me.” They seldom grabbed students for a learning opportunity or medical procedure. Requests made by students to observe and participate in medical conditions and procedures were often discouraged and dismissed. One student observed:

They overheard a group of residents speaking, like, poorly about things like “They're so annoying they won't leave me alone” and “I don't get why they're so stupid.” This is obviously something I heard from someone else, but I know it stuck with them, and really discouraged them from that specific specialty. Um, I mean, there are stories that go around the school, unfortunately. (P11)

Various instances related to dysfunctional medical teams were reported. The students described a lack of trust and respect between attendings and nurses within some medical teams, individual residents were excluded and ignored intentionally, and leaders exhibited poor leadership qualities such as micromanagement and lack of direction. Dysfunctional medical teams led to a variety of issues that hindered role modeling, team effectiveness, and overall satisfaction. As one student described in the following:

There’s one surgeon that is not incredibly well-liked, um, by different, like nurses and things like that, so, like, every time he would leave after the surgery, the whole room would just start complaining about him “Thank you for being our buffer so he doesn't yell at us too much today” ... and it was also really awkward for me as, like, the medical student who was assigned to his service for the day. I knew he was giving me my evaluation. It just kind of put you in that weird position constantly, smiling and laughing along with whatever anybody in the room is saying. (P9)

Clinicians, residents, nurses, and even patients sometimes demonstrated a lack of sensitivity to all individuals regardless of group status. They made comments and showed explicit or implicit biases towards people based on their ethnicity, weight, gender, race, sexuality, age, social-cultural background, or status (medical students vs. physicians). For example, one student reported that an attending felt that “being transgender was a mental illness” (P5), and another interviewee described how patients treated “my fellow female cohort in a different way that I get treated as a guy in med student” (P2). The following student elaborated on her experience:

I’m coming from a culture of, uh, I’m, we're more conscious about, like, um, ranking [social hierarchy]. He kind of assumed that because of the background I came from, that is why I'm quieter, um, and he put in a comment [in my evaluation], like, he's trying to make it a positive thing, but it can be spun in a different way. The person who's reading it is saying, you know, I was not outspoken, but that can be misinterpreted as me not knowing my information. (P4)

#### Potential Causes of Unprofessionalism and Bias

Potential factors that contributed to unprofessionalism and bias emerged as an evident theme. The participants identified various factors that contributed to unprofessionalism and bias within the medical field: societal, local, and individual. While the students perceived unprofessionalism and biases were related to individual personalities and perspectives, they reported the influence of societal factors and local contexts. At the societal level, the participants recognized that broader societal influences played a role in shaping attitudes and behaviors including professional norms and systemic hierarchy that may perpetuate unprofessionalism and bias within healthcare settings. For instance, the students reported how orthopedics tended to be a male-dominated field and pediatrics female-dominated; as such, “there were definitely comments from the surgeons and they seemed to really question if I [as a female] knew what the lifestyle was like” (P5). The students also described that they were fully aware of the hierarchy in medicine, as stated “when you have attendings, who traditionally bring more money to the hospital, and have a higher status, no one really wants to go against them, due to their leverage” (P1).

At the local level, the participants acknowledged the impact of the specific clinical environment such as organizational culture, the employee structure regarding diversity, equity, and inclusion (DEI) representation, institutional structure, and the dynamics within their immediate healthcare teams. The students observed that CMED had different cultures and norms across different clinical locations. One student reported that she felt fortunate and “privileged” because the location where she rotated had a significant number of surgeons who were women of color and a diverse representation among residents and patients. These local factors, including the demographic makeup of the team and patient population, had a notable influence on interactions and played a role in shaping perceptions and behaviors related to professionalism.

#### Impacts on Learning and Well-being

The focus group discussions discussed various impacts of unprofessionalism and biases on performance, learning opportunities, well-being, and trust. The participants reported that the presence of unprofessionalism and biases negatively affected their performance. They recognized that these issues had significant consequences in various aspects of medical education and practice. Unprofessional behaviors and biases hindered learning opportunities for students, interfered with their confidence and decisions to pursue certain specialties, and discouraged them from seeking appropriate guidance, mentorship, or constructive feedback. Unprofessionalism and biases impacted their ability to focus, perform at their best, and fulfill their potential as future healthcare professionals, as stated by one student in the following:

I think normally I’m really excited to go to rotations, and that was the first time that I kind of felt like dreading going because, out of fear, that I would mess up and somehow get yelled at. I just felt like they didn’t want me there…therefore it kind of left me feeling down some days. I wasn't super excited as I normally would be to go to my next shift. I was more hesitant to ask questions or that type of thing…I didn’t volunteer to participate, and I missed clinical opportunities because of that.” (P6)

The students reported that unprofessionalism and biases significantly impacted their well-being and trust in the medical profession. They felt demoralized, anxious, or disengaged in an environment where they were subjected to unprofessional behavior or biased treatment. These negative experiences made them question the opinions and attitudes of some healthcare providers. As stated in the following, the participant felt a sense of unease about the health profession.

I think I had anxiety, like, knowing that I would have to work with that provider who had said these things, but also a bigger picture, I think it made me question some of the opinions that providers might have, made me feel nervousness with any providers that they might be thinking these things or that if I were to be honest about my opinions… it just impacts the trust I have in the health care system, from a larger perspective. (P5)

## Discussion

The quantitative results revealed that a significant portion of students, approximately one-third, believed there were frequent disconnects between the level of professionalism demonstrated by faculty and what they were taught. Additionally, they reported the occurrence of unprofessional behaviors displayed by nurses and residents, albeit less frequently. The quantitative data also highlighted the presence of explicit and implicit biases related to race, religion, ethnicity, gender, and socioeconomic status. These findings aligned with the observations from the focus group participants, who indicated that faculty, residents, and nurses exhibited a lack of respect, compassion, commitment to professional teaching responsibilities, and collegiality within the team. The qualitative results provided further insights into the biases experienced by students. The biases identified encompassed various aspects such as ethnicity, gender, race, sexuality, age, and social-cultural background, which was consistent with the quantitative findings. The quantitative result found the existence of unprofessional behaviors exhibited by their peers; however, the interviewees did not extensively discuss this aspect, likely due to the nature of the focus group discussions where the participants may not have felt comfortable speaking openly about their peers’ behaviors.

The quantitative data revealed that approximately 20% of the M3 participants and 11% of the M4 participants disagreed or strongly disagreed that their clinical assessments were solely influenced by their performance. These findings were consistent with the qualitative findings discussed in the focus groups. In addition, the participants in the focus groups concluded that unprofessionalism and biases had far-reaching consequences on their learning, well-being, and trust beyond just their performance. These results align with findings in the existing literature [24]. When students encounter such behaviors, they feel marginalized, excluded, or dismissed, which interfere with their specialty choice, limit their access to valuable educational experiences, and impede their professional growth. Most significantly, unprofessionalism and biases erode the trust that students had in their educational environment. When they witness or experience unprofessional behavior or bias, it undermines their confidence in the fairness and integrity of the medical system.

Furthermore, the focus group results highlighted that unprofessionalism and bias in the medical field could not be attributed solely to individual factors. The finding emphasized the interconnectedness of societal, local, and individual influences. The participants discussed how societal norms, professional beliefs, and medical hierarchy could seep into the medical field, leading to discriminatory practices or biases based on race, gender, ethnicity, religion, socioeconomic status, and other factors. They also noted that different clinical settings and institutions had their own unique cultures and norms, which could either facilitate or hinder professionalism and unbiased practices.

Taken together, the results from both the quantitative and qualitative data demonstrate the presence of unprofessionalism and biases within the current medical education environment, which have detrimental impacts on performance, learning, and well-being. These results are consistent with the previous literature [6, 16, 25]. The pattern of unprofessional conducts and biases about the historical poor treatment of medical students is not a recent phenomenon but has persisted across generations within medical education. The reasons behind unprofessionalism and bias include hidden, unspoken norms engrained and propagated by generations of physicians, influences from society, and the local environment, all of which extend beyond personal attributes. These findings indicate a need for comprehensive interventions and reforms to address these issues, including targeted education and training on professionalism and cultural competence, reflecting on cultural norms and stereotypes, fostering a respectful and inclusive learning environment, and promoting effective communication and collaboration among all stakeholders [26]. It is essential for medical schools to adopt strategies that prioritize psychological safety, establish preventative action plans, increase reporting of negative behaviors, and evaluate the professionalism curriculum. Raising awareness of biases and harm that may be experienced by learners is crucial for enhancing educators’ behavior and cultivating an inclusive and positive learning environment. Mentoring has proven to be effective in improving faculty reporting, boosting confidence in institutional handling of mistreatment reports, enhancing career satisfaction, and improving retention of faculty [27, 28]. These positive outcomes can easily extend to students and residents. To ensure continuous professional development, support should be provided for Justice, Equity, Diversity, and Inclusion (JEDI) initiatives and offer restorative justice and affirmation training for clinical educators. Additionally, when healthcare teams and patients represent a range of backgrounds and experiences, it fosters a more inclusive and respectful culture, leading to improved interactions and heightened awareness of biases. By doing so, medical education programs can create a more equitable, inclusive, and professional learning environment for all students optimizing learning opportunities while enhancing a growth mindset, and student well-being, and fostering a sense of trust. Ultimately, it will produce competent, compassionate healthcare professionals while promoting culturally sensitive and professional medical educators.

### Limitations

This study was conducted on a small scale within a single college, involving two cohorts with a low response rate from the M4 students. While considerable efforts were made to recruit participants for surveys and focus groups, it is crucial to acknowledge that students may have concerns about potential retaliation, which could affect their willingness to openly share all of their perceptions and experiences. Consequently, caution must be exercised when generalizing the results to other medical schools. Future studies may also investigate how many students changed their choice of specialty based on the impact of the learning environment. Furthermore, there are limitations on self-report perception surveys, and it is difficult to reconcile the students’ opinions about the validity of their clinical assessment. One thing to consider would be self-reflection on clinical vignettes with faculty feedback to strengthen understanding and validity of the information. Multiple perspectives from health professionals are needed to thoroughly understand unprofessional behaviors and biased perceptions. To gain a comprehensive understanding of unprofessionalism and biases in varying contexts, further studies involving multiple medical schools are necessary.

## Conclusion and Implications

In conclusion, the study acknowledged the existence of unprofessionalism and biases and found the detrimental effects on students’ performance, learning, and well-being within the research context. The study provides important implications for the CMED Learning Environment Committee and beyond. It is vital to create a learning environment that prioritizes diversity, equity, and inclusiveness and evaluate the effectiveness of professionalism curriculum. Addressing unprofessionalism and bias requires a collective effort to challenge stereotypes and biases, promote awareness and understanding, provide education and training, and establish clear expectations for professional conduct in faculty evaluations. It requires comprehensive approaches that encompass systemic reflections, institutional policies, and personal reflection and growth. Considering the recent Supreme Court ruling on race in college admissions, this study calls for more analysis of the complex decision to determine how it translates into institutional policies and practices.

Overall, by acknowledging and actively working to mitigate unprofessionalism and bias in the healthcare learning environment, medical schools and affiliated clinical settings can create an environment where medical students are inspired to embody the values of diversity, equity, and inclusiveness and aspire to high standards in practice. Therefore, they can incorporate these values into their future practice as healthcare professionals, challenge biases and stereotypes that hinder the delivery of equitable healthcare, and navigate complex psychosocial factors and cultural influences to shift the current culture in patient care.

## Appendix. The focus group semi-structured protocol

1. (Show Central Michigan University College of Medicine EPOs to participants). The first question focuses on witnessed and experienced behaviors and comments demonstrating a lack of professionalism.
  - Regarding the question “there are disconnects between what I am taught about professional behavior and the level of professionalism that is demonstrated by faculty”, could you provide specific examples? In other words, what were specific behaviors, attitudes, and statements made or demonstrated by faculty that were inconsistent with what you were taught in school?
  - How about unprofessional behaviors demonstrated by resident, nursing, and your peer students? Could you provide specific examples?
2. The next question is related to bias
  - Could you provide examples of explicit and implicit bias regarding gender, race, ethnicity, and socioeconomic status? On you? On other students? Or other people? Provide examples of derogatory, sexist, offensive comments/insults/remarks made by faculty, residents, and/or nursing.
3. How do you think unprofessional behavior and bias by faculty and residents (e.g., gender race, ethnicity, and socioeconomic status) can influence your clinical performance/evaluations/grades? Your wellbeing? And any other areas?
4. Any additional comments?
